# Supplementary figures and images for: Distinct populations of crypt-associated fibroblasts act as signaling hubs to control colon homeostasis
Source: PLoS Biol. 2020 Dec 11;18(12):e3001032. doi: 10.1371/journal.pbio.3001032 (PMC7758045; doi:10.1371/journal.pbio.3001032)

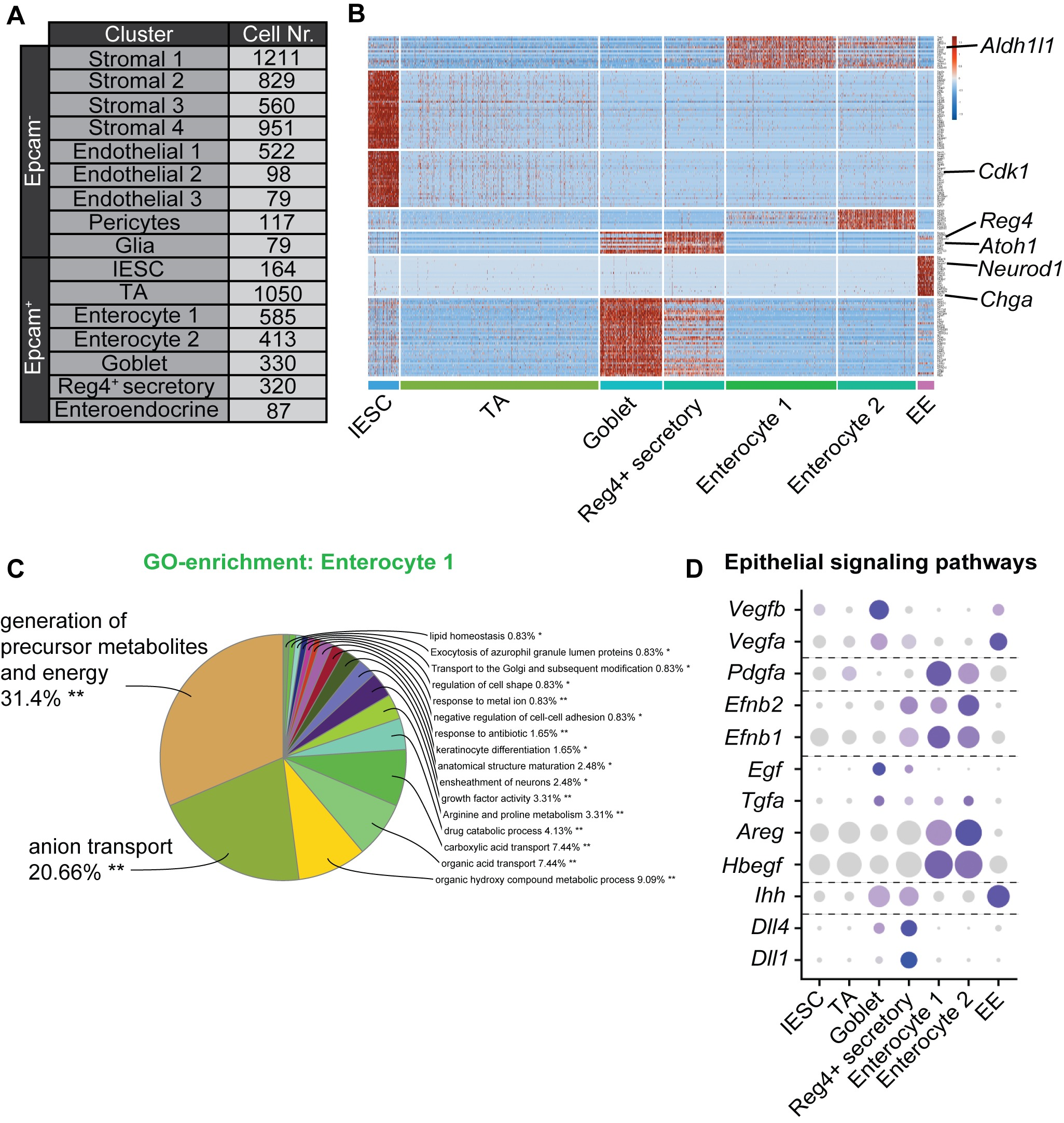

Supplement: S1 Fig — (A) Cell numbers of the particular clusters indicated in Fig 1C. (B) Heatmap of genes that are differentially expressed among the epithelial clusters (0.97 quantile), reveals heterogeneity between and similarity within the major epithelial lineages (stem-/transit-amplifying-, secretory-, absorptive-, enteroendocrine cells). (C) Relative expression of epithelial signaling pathway components. (Dot plot, size, and color of the dot represent the percentage of cells which express the transcript and the average expression level within a cluster, respectively). (D) GO enrichment terms for Enterocyte 1. GO, Gene Ontology. (TIF) [file pbio.3001032.s001.tif]

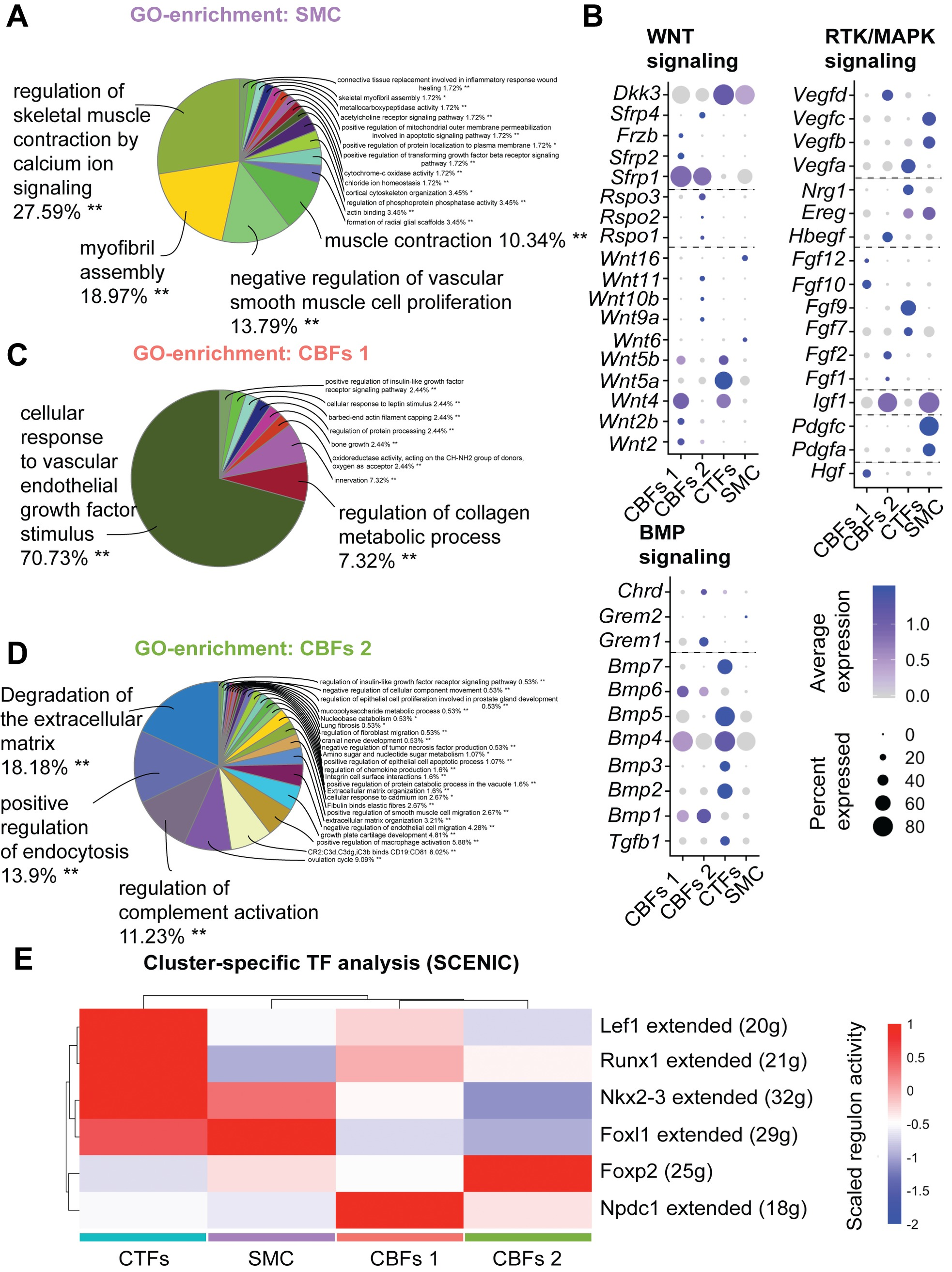

Supplement: S2 Fig — (A) GO enrichment terms for SMC. (B) Relative expression of factors involved in WNT, BMP, and RTK/MAPK pathways. (Dot plot, size, and color of the dot represents the percentage of cells which express the transcript and the average expression level within a cluster, respectively). (C) GO enrichment terms for CBFs 1. (D) GO enrichment terms for CBFs 2. (E) Heatmap showing the specific enrichment for transcription factor regulon activity in specific stromal clusters (scaled regulon activity, SCENIC). Bmp, bone morphogenetic protein; CBF, crypt-bottom fibroblast; GO, Gene Ontology; MAPK, mitogen-activated protein kinase; RTK, receptor tyrosine kinase; SCENIC, single-cell regulatory network inference and clustering; SMC, smooth muscle cell. (TIF) [file pbio.3001032.s002.tif]

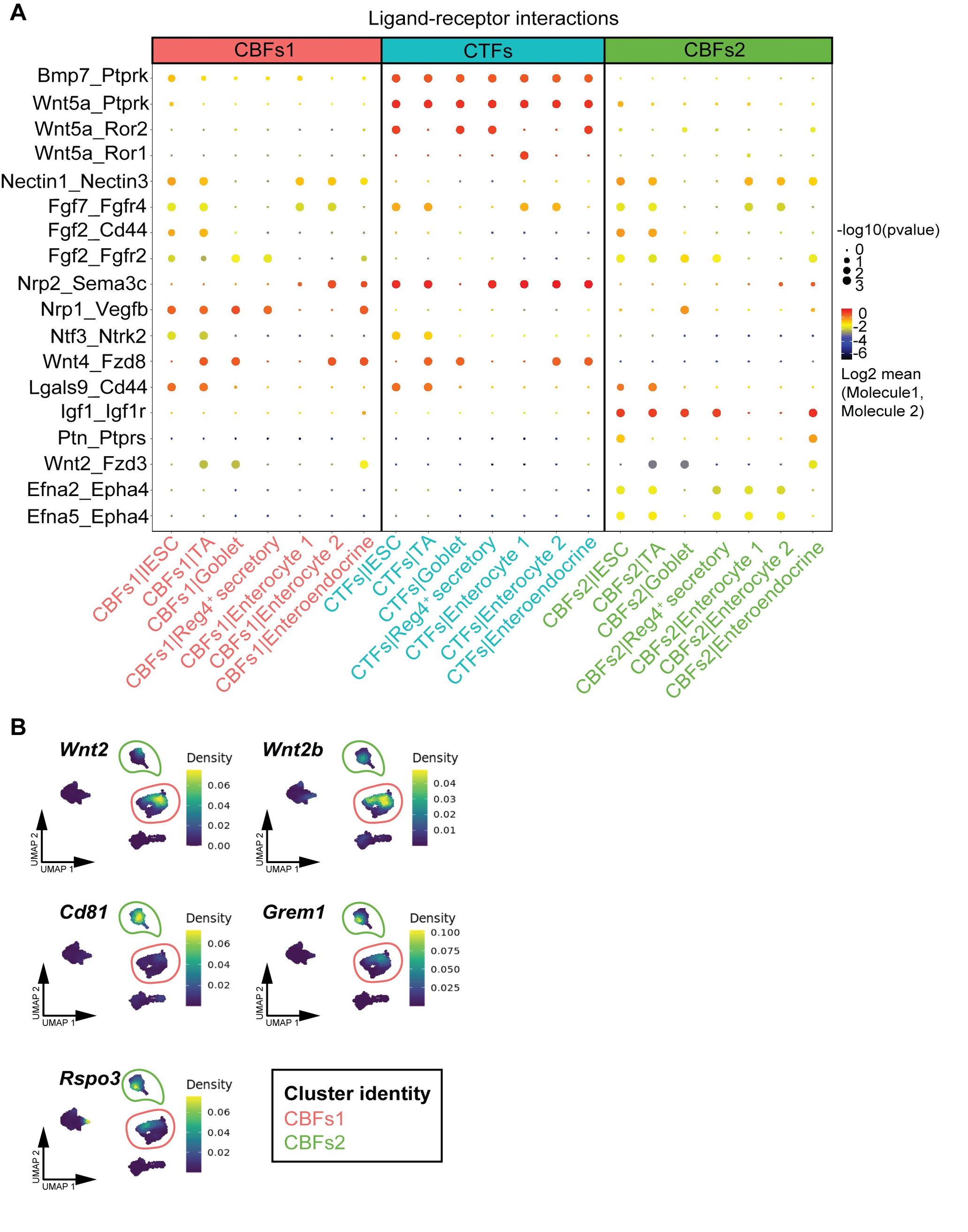

Supplement: S3 Fig — (A) Putative ligand–receptor interactions between CBFs1, CBFs2, CTFs, and colonic epithelial subpopulations. Size of the dots represents the significance of the interaction, and color shows the expression of the ligand and receptor in the interacting cell types (CellphoneDB). (B) Density of Wnt2, Wnt2b, Cd81, Grem1, and Rspo3 expressing cells. (UMAP, color indicates the density of cells expressing transcript). CBF, crypt-bottom fibroblast; CTF, crypt-top fibroblast; UMAP, Uniform Manifold Approximation and Projection. (TIF) [file pbio.3001032.s003.tif]

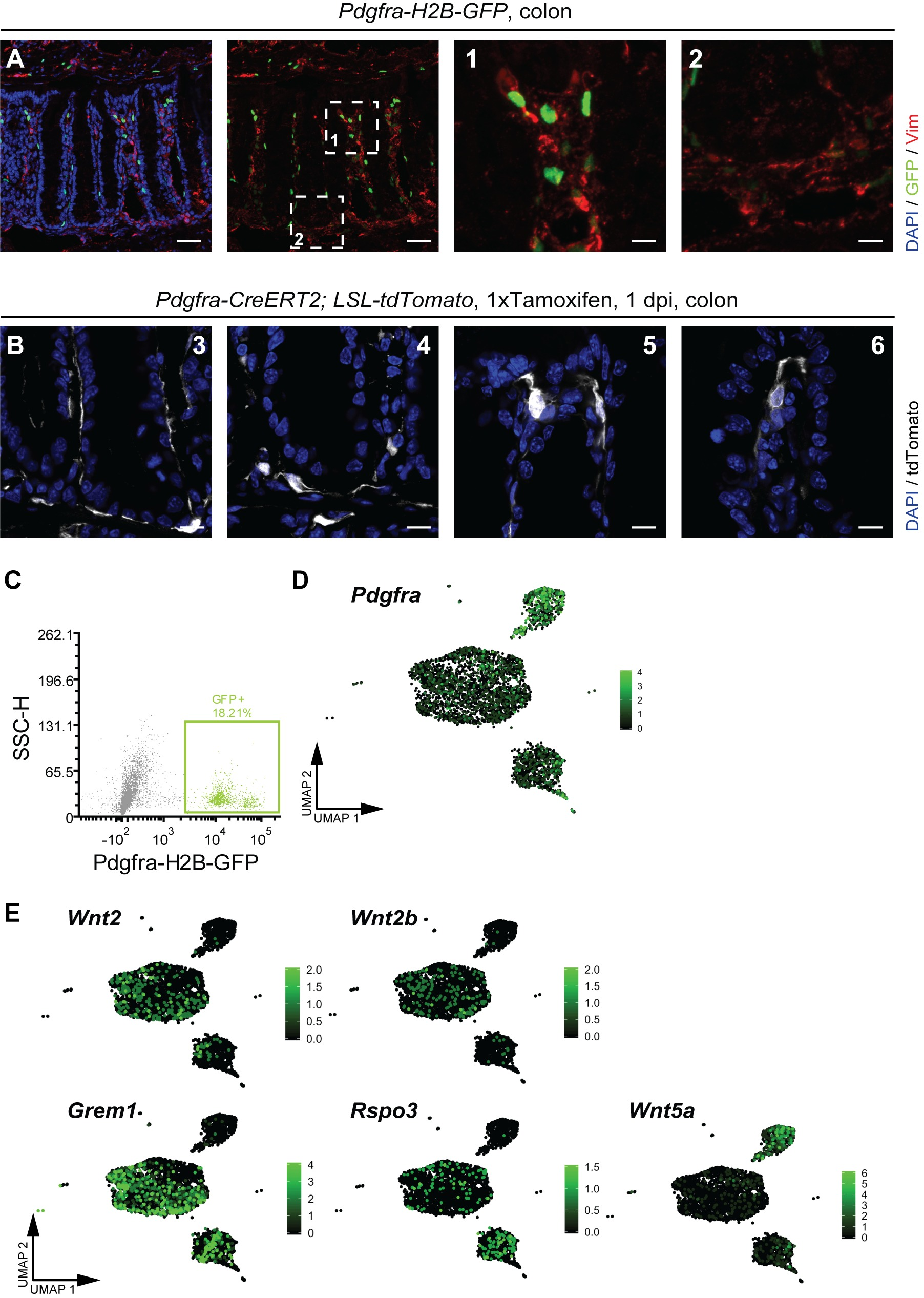

Supplement: S4 Fig — (A, B) Cryosections of Pdgfra-H2B-eGFP mice (A) and Pdgfra-CreERT2; LSL-tdTomato mice (B). (A) Vimentin expression (red) in Pdgfra+ cells (green) on representative Pdgfra-H2B-eGFP colonic tissue sections confirms their fibroblast identity. (Scale bar = 40 μm) (1,2) Insets of crypt top and crypt bottom, respectively (Scale bar = 5 μm). (B) Pdgfra-CreERT2; LSL-tdTomato lineage tracing (single tamoxifen injection, 1 d.p.i.) reveals morphology of Pdgfra-expressing cells, tdTomato (white) (Scale bar = 10 μm) (C) Flow cytometry analysis for GFP of a mesenchymal single-cell suspension isolated from Pdgfra-H2B-eGFP mice. (Raw data: S5 Data) (D, E) Relative expression of Pdgfra (D) and colonic niche factors (E) in Pdgfra+ colonic mesenchymal cells. (UMAP, single cells are colored according to transcript expression). CBF, crypt-bottom fibroblast; CTF, crypt-top fibroblast; d.p.i., days post injection; Pdgfra, platelet-derived growth factor receptor A; UMAP, Uniform Manifold Approximation and Projection. (TIF) [file pbio.3001032.s004.tif]

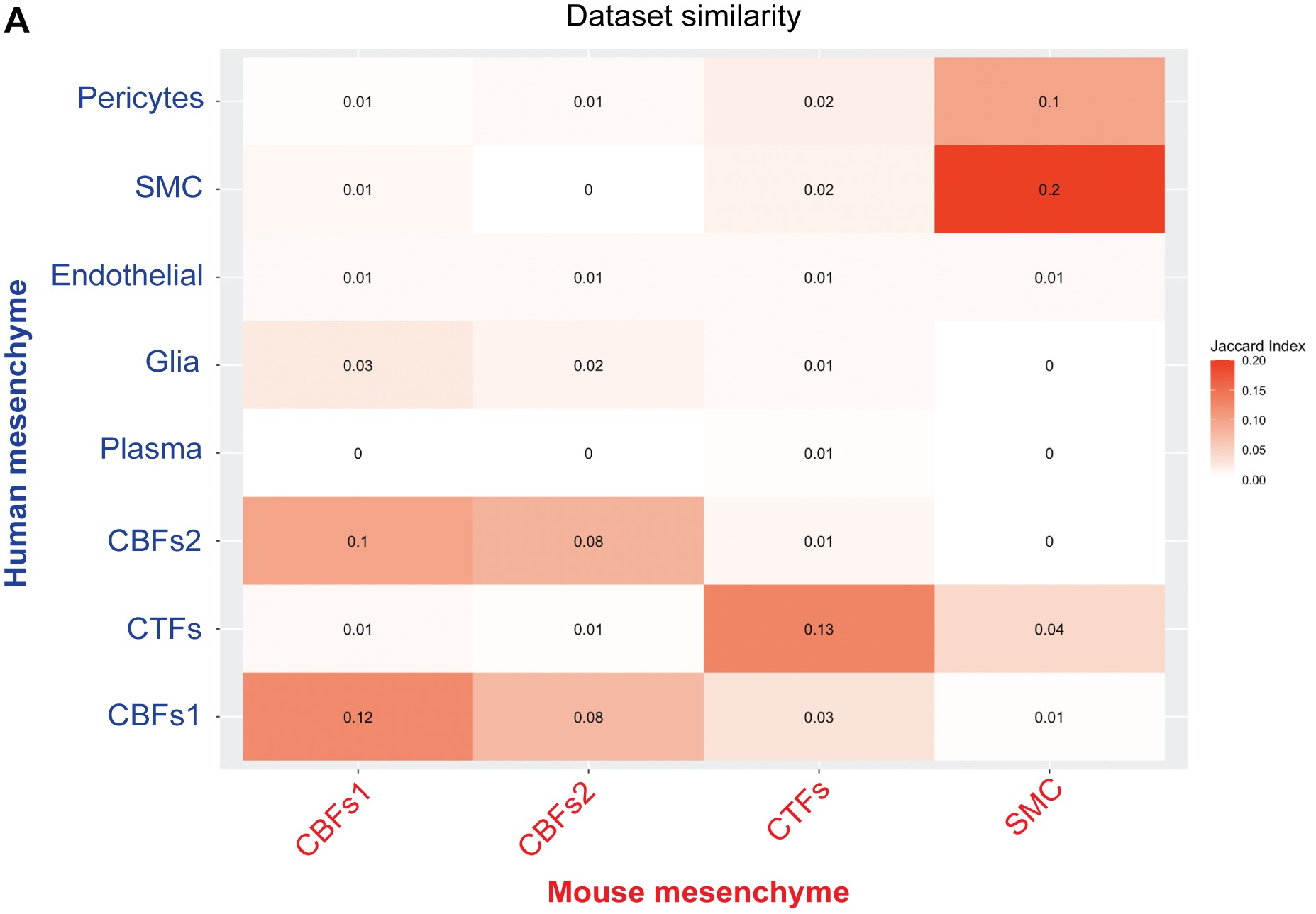

Supplement: S5 Fig — (A) Heatmap showing the similarity (Jaccard index) of murine and human colonic mesenchymal subpopulations (matchSCore2). (TIF) [file pbio.3001032.s005.tif]
