# Supplementary material for: Distinct populations of crypt-associated fibroblasts act as signaling hubs to control colon homeostasis
Source: PLoS Biol. 2020 Dec 11;18(12):e3001032. doi: 10.1371/journal.pbio.3001032 (PMC7758045; doi:10.1371/journal.pbio.3001032)
Supplement: Attachment — Submitted filename: PLOS_biology_Response_to_reviewers.pdf [file pbio.3001032.s011.pdf]

We would like to thank all reviewers for their questions and valuable comments. We are very pleased that they share our excitement about the findings and our estimate of the value of our dataset for the research community.
All comments are addressed point-by-point below. Following the reviewer's recommendations, we tried to improve and clarify some figures and associated text.
The main changes are:

- the addition of a paragraph commenting on the differences between the subpopulations CBFs1 and CBFs2 (including supporting data on new supplementary data: S3 Fig).
- the addition of a new figure showing ligand-receptor interactions (S3 Fig)
- a new figure describing the morphology of Pdgfra-expressing colonic mesenchymal cells (S4 Fig)
- we added S5 Fig, which displays the interspecies conservation between colonic mesenchymal cells, based on the entire transcriptome, strengthening the translational value of our observations.

The minor text changes throughout the manuscript that serve to improve the clarity of the portrayed messages can be traced by track changes (and are additionally highlighted in green).

Rev. 1:

In this manuscript, Brügger et al. characterize the epithelial and mesenchymal/fibroblast populations of the mouse colon using sc-RNAseq. They compare this to the human colon cell populations and find they are quite comparable. The story is descriptive (which I do not intend to sound like a bad thing) and results in the identification of three different populations of fibroblasts: two associated with the bottom of the crypt (CBF1 and CBF2) and one associated with the top of the crypt (CTF). These populations express different signaling factors that either promote stemness (CBF: Wnt2, Wnt2b and Rspo3) or differentiation (CTF: Wnt5a and Bmps). The authors also provide evidence that these populations correspond to spatially separated Pdgrfa+ populations in situ (Pdgrfa-high: CTF, low: Pdgrfa-CBF1 and 2).

The manuscript is clearly written, with a balanced and focused introduction. The dataset (>7000 cells total, >3000 transcripts per cell) has been deposited at NCBI GEO and promises to be a useful resource for scientists interested in the interaction between intestinal stem cells and their presumed niche.

This is a straightforward study and I only have a few remarks that perhaps the authors can consider to clarify a few points:

1. The introduction mentions that most other studies use specific cell isolation protocols. It is not clear what the authors refer to, given that they themselves of course also use an enzymatic digestion and cell isolation protocol (i.e. FACS sort for EPCAM+ and EPCAM- cells). I think I get what they mean (theirs is 'just' a sort for epithelial versus non-epithelial), rather than a specific enrichment for a subpopulation, but perhaps they can clarify this a bit.

We have adjusted the text to clarify this point. While indeed we use protocols specifically aimed at isolating both epithelial and non-epithelial cells in our study, we sort epithelial (EPCAM+, CD45-) and non-epithelial (EPCAM-, CD45-) cells originating from both isolations (i.e. epithelial and non-epithelial cells isolated from the epithelial fraction after, and remaining epithelial and non-epithelial cells isolated from the fraction after the majority of epithelial cells was removed). Therefore, we make sure not to lose

e.g. non-epithelial cells that closely interact with epithelial cells or vice versa, that were hitherto potentially lost when looking for example only at epithelial cells. Such a “pooling approach” is one of the benefits of our study, since it was not done so far.
(The respective sections were changed in the manuscript, both in the introduction and results part, to describe it more thoroughly)

2. In Figure 1B, does this refer to the input prior to sorting or is this purity after sorting?

Figure 1B refers to the gating strategy chosen prior to sorting (information clarifying it was added to manuscript).

3. Related to the sorting: Can the authors comment on and/or quantify the ratio of these different cell populations (CBF1/2 vs CTF vs epithelial cells)?

The following numbers are based on the overall sorting numbers and ratios observed in the single-cell data and refers to the isolated cells:
91.2% are epithelial cells and 8.8% are mesenchymal cells. This information has been added to the manuscript. However, the majority of the lamina propria smooth muscle cells, are not dissociated by our enzymatic treatment and thus underrepresented.

CTFs make up 1.6% and CBFs1 and CBFs2 make up 2.4% and 1.6% of the isolated cells, respectively.

4. Figure 2 and throughout: Can the authors speculate on the difference between the CBF1 and CBF2 population? Are there any discriminatory marks that separate these two otherwise functionally overlapping populations? Based on 2G this seems a logical step to include in this manuscript and something the authors should be able to do with the tools and analysis pipeline at hand, or is this something the authors plan to follow up on later? Alternatively, could it be some sort of experimental artifact? A list of differentially expressed genes in these populations might be informative.

This is a very important comment. The paragraph concerning the difference between CBFs1 and CBFs2 has been added to the manuscript.

5. From the legend of Figure 3A-C it is not clear that we are looking at cryosections, but I imagine that this is what is depicted, based on the methods?

The Figure 3A-C depict cryosections of fixed frozen tissue. The legend has been updated accordingly.

6. Related to 5: Has the GFP signal been corrected for DAPI? The authors describe in the methods how they performed the GFP quantification, but this seems to related to masking of nuclei to include. Ideally, the GFP signal is corrected for the DAPI intensity to make up for depth/sectioning/processing/imaging differences at different locations on the slide. If it is not done or cannot be done, this should be noted in the legends and/or methods.

While we did not correct the GFP signal for DAPI, as described in the methods, we excluded all the nuclei with only partially sectioned/dim nuclei (low quality) and made

sure that only nuclei that were fully capture in the Z-stack were considered for the final analysis. To clarify this, we added more detail to methods section.

Rev. 2: Shalev Itzkovitz – this reviewer has waived anonymity

In this work, Brugger et al. perform single cell RNAseq experiments to characterize the mesenchymal cell types constituting the colonic crypt niche. They uncover three distinct Pdgfra+ fibroblast populations, expressing distinct antagonistic morphogens. More specifically, crypt bottom fibroblasts, which express canonical Wnt ligands, Rspo3 and Bmp inhibitors, and crypt top fibroblasts, expressing non-canonical Wnt ligands and Bmp ligands. The study is well performed and will be an important resource for biologists interested in stem cell biology. The following points should be addressed in a revision:

- The supplementary figures nicely show the expression of ligands of major signaling pathways among the three fibroblast populations, however the paper would be strengthened by a more comprehensive analysis of ligand-receptor interactions, most importantly between the fibroblasts and epithelial compartments. Specifically, what are the main elevated epithelial ligands expressed in tip/crypt enterocytes with matching elevated tip/crypt fibroblast receptors and vice versa? There are several examples, e.g. Pdgfra in the crypt tip enterocytes and Pdgfra in the CTF, Rspo3 in CBF and matching epithelial receptor Lgr5 at the crypt bottom. Such analysis can be performed by tools such as https://www.cellphonedb.org/ or NicheNet (https://www.nature.com/articles/s41592-019-0667-5). Similarly, are there interactions between the fibroblast populations and pericytes/endothelial clusters?

Following this valuable suggestion, we ran the CellphoneDB algorithm on our data. We were able to confirm known interactions, such as Wnt2 from CBFs1 interacting to Fzd3 in epithelial cells or Wnt5a from CTFs signaling to Ror1 and Ror2 in epithelial cells. In addition, we discovered potentially interesting new interactions, such as Igf1 from CBFs2 signaling to Igf1r in epithelial cells or Fgf2 from CBFs1 and CBFs2 signaling to Fgfr2 and Cd44 in epithelial cells. We have provided this new data as a new S3 Fig.
However, given the current (lack of) understanding of colon crypt zonation we cannot extract the information to answer what are possible ligand/receptor interactions between, for example, crypt tip enterocytes and fibroblasts. We cannot simply assume that the same markers that were found in villus zonation of enterocytes in the small intestine are accordingly distributed in colonic enterocytes in the same way. Once data on colon crypt zonation become available our dataset could be interrogated to investigate this interesting question.

- Can the authors comment about the potential spatial differences between CBF1 and CBF2? Is one population closer to the epithelium? Do any of these populations overlap trophocytes (https://www.nature.com/articles/s41556-020-0567-z)?

In contrast to the situation in the small intestine, in the colon all Pdgfra^{low} cells - CBFs1 and CBFs2 - were located in close proximity to the crypt/stem cell compartment: 1-3 cell diameters (Fig 3A). Looking in more detail into the different spatial localization of CBFs1 and CBFs2 is hampered by the fact that most marker genes/differentially expressed genes do not show clear black/white patterns of expression within the colonic stromal cell populations. As a result, the selection and analysis of potential

antibody/smFISH staining proves difficult and is uncertain to yield further insight into the subject.
Based on a comparison of the scRNAseq data, CBFs2 show stronger overlap to the trophocytes described by McCarthy and colleagues for the small intestine. For example, high expression of the marker gene *Cd81* and the Bmp antagonist *Gremlin1* are shared characteristics. These observations have been added to the manuscript when discussing the difference between CTFs1 and CTFs2, and corresponding data added to S3 Fig.

- Do CTFs express Lgr5, as has been shown for villus tip telocytes (VTTs, ref 20)? The authors should comment on the overlap and differences in gene expression between CTFs and VTTs, which express many of the discussed CTF markers, such as the non-canonical Wnt5a, Bmp ligands and F3.

Indeed, a low number of cells in the CTFs cluster express Lgr5.

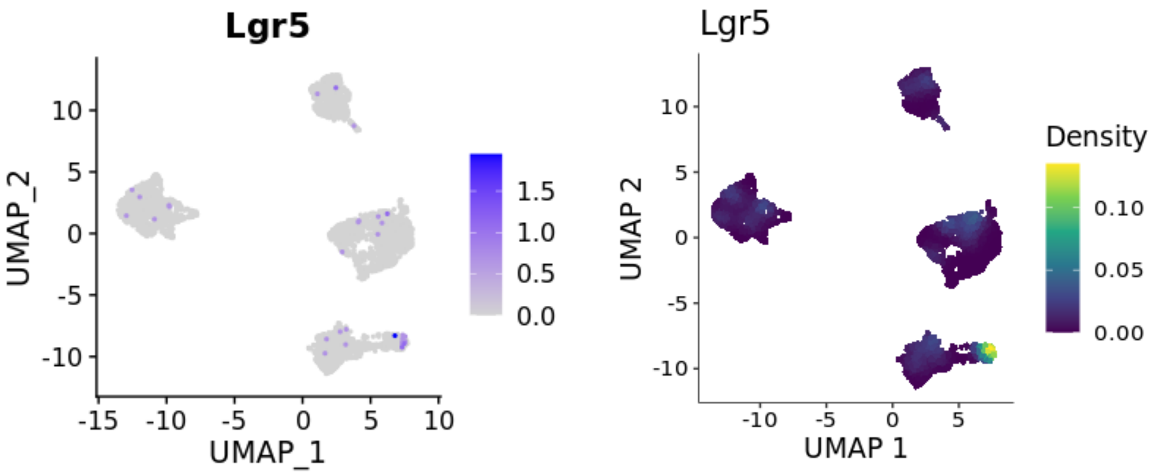

Moreover, many of the factors that are expressed in VTTs are shared with CTFs, such as Wnt5a and Fn3, and high expression of Bmp2 and Bmp4. However, there are differences; several interesting genes expressed in VTTs have a different pattern: the ligand Egf is not expressed in CTFs and microfibrillar genes, such as *Mfap5, Emilin2* and *Fbn1*, are more highly expressed in CBFs1 and CBFs2 in the colon.

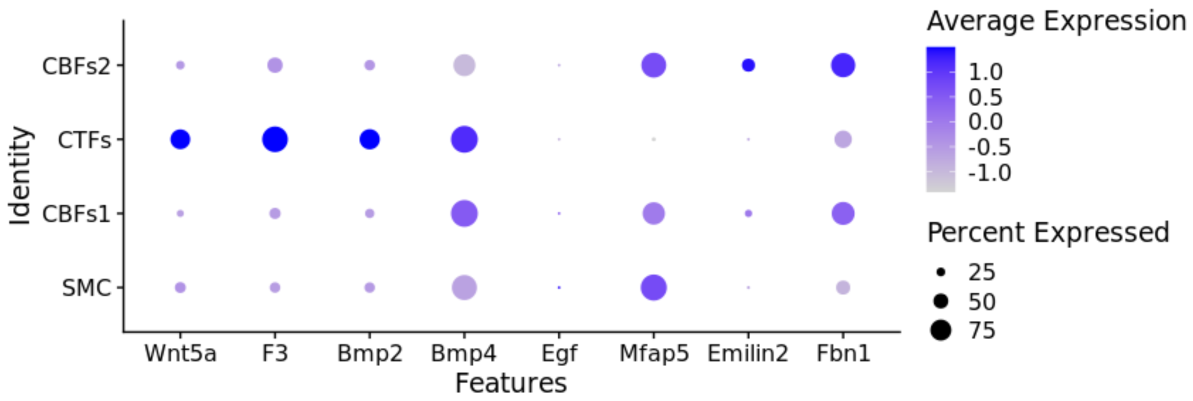

-It would be informative to add immunofluorescence for PDGFRA protein, which labels the entire cell bodies. Telocytes have a unique morphology with elongated processes that 'wrap around' the crypts, as well as tiny 'telopode' protrusions into the lamina propia, such staining for PDGFRA would reveal such morphologies in the colon.

The antibody staining for PDGFRA protein can be found in Figure 3A. However it does not label the entire cell bodies, probably corresponding to the distribution of the protein. More information about the morphology of Pdgfra positive cells can be learned from pictures of *Pdgfra-CreERT2; LSL-tdTomato* mice - tdTomato labels the entire cell. This data has been added to S4 Fig.

- The expression of both the canonical Wnt ligands and antagonists is intriguing, do the same CBF cells express both ligands and antagonists, or are they expressed in different CBF subsets?

This question is not so easy to answer due to general issues with dropout rate in single-cell RNA sequencing and the resulting uncertainty whether low expressed genes, such as Wnt ligands are possibly expressed in all cells of the clusters and simply not captured or whether they are really only expressed in a subset of the cells. However, we queried the co-expression the canonical Wnt ligand *Wnt2b* and Wnt signaling inhibitors (*Sfrp1, Sfrp2, Dkk3*) and found rare cells that co-express both (yellow cells in graph).

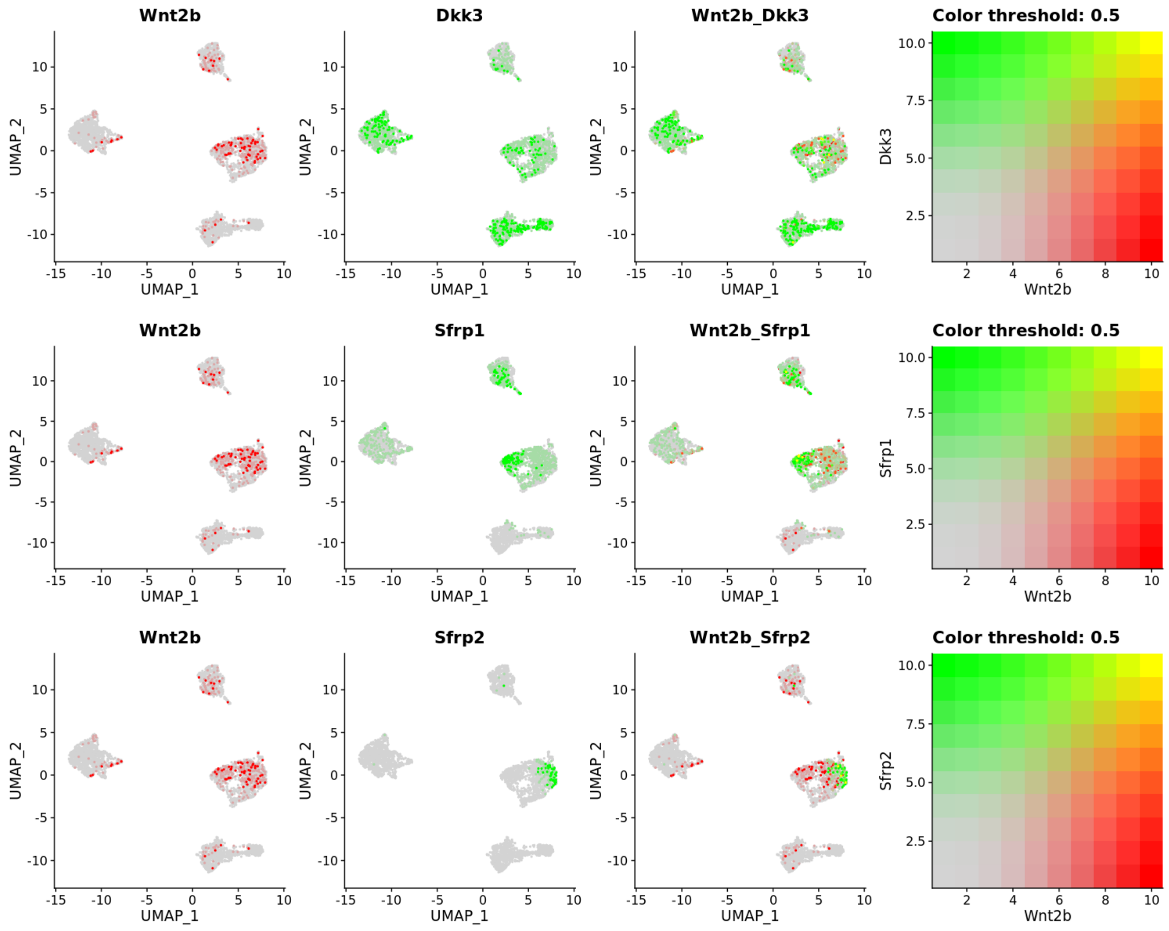

- Row 213 - add ref for the connection between the Lef1 regulon and Lgr5 expression.

The references were added to the manuscript and wording slightly changed. *Lef1* is both a Wnt/b-catenin signaling transcription factor, as well as a canonical Wnt target gene itself. Given that *Lgr5* is a canonical Wnt target itself the expression of *Lgr5* and the enrichment for the *Lef1* regulon in crypt-top-fibroblasts point towards the same possibility – a putative canonical Wnt signaling activity in crypt-top-fibroblasts.

- Row 406 - add ref. for "McCarthy and colleagues"

The reference was added accordingly.

Rev. 3:

Summary of opinion: The instructions to the reviewer start "Short Reports should be novel, provocative and of general interest". The findings here are of general interest and high quality. They are not particularly novel, and because they support and extend prior studies, they are only modestly provocative. Overall, this manuscript contributes to the research field of scRNAseq and stem cell regulation, revealing the heterogeneous cell populations that regulate cellular homeostasis in the colon. Given the useful and high quality confirmatory, rather than novel, nature of the manuscript, I defer to the editor to decide if it meets the journal's editorial guidelines.

General comments: Brügger et al. aim to identify mesenchymal cell populations regulating proliferation and differentiation of colonic stem cells located in the crypt base. To look into the heterogenous nature of the intestinal mesenchymal tissue, scRNAseq was performed. The authors identified three distinct PDGFRA positive fibroblast subpopulations expressing Wnt or Bmp signals that regulate stem cell fate.

Generally, the setup of experiments is comprehensible and the authors present their data in a logic and conclusive way. The experiments and the interpretation of the data appear reliable and solid. The cell number (7395) and the read depth (3243/cell) is robust and will be a useful resource.

However, in a recent study published by McCarthy and colleagues (and cited appropriately) nearly identical results were presented. Here, a very similar experimental setup was used to identify mesenchymal cell populations regulating stem cells in the small intestine. McCarthy et al found the same cell populations with very similar gene expression profiles and localizations within the intestinal tissue. Therefore, this study by Brüegger et al has largely a confirmative character, since it reflects and verifies the results of the study from McCarthy and colleagues and doesn't break much new ground.

Additionally, by reanalysis of a recently published dataset, Brüegger et al could show that similar cell populations exist in human colonic tissue what might make this study useful for researchers focused on tumor initiation and progression.

Overall, this manuscript contributes to the research field of stem cell regulation revealing the heterogeneous cell populations which fulfil the regulation of the sensitive cellular homeostasis in the colon.

Major points:

CBF1 and CBF2 were shown to represent 2 distinct cell populations. However, the authors did not show data about the spatial localization of both cell types within the tissue.

This is an interesting point, however one that we are not able to practically address, since there is no suitable set of markers to define the localization of the two populations. Moreover the data in Fig 3A shows that Pdgfra^{low} cells (CBFs1 and CBFs2) are located in close proximity to the crypt/stem cell compartment (1-3 cell diameters). Without the possibility to isolate the cells and examine the functional relevance a staining of higher resolution is not more informative. That said the suggestions of the reviewer are excellent ideas for future work.

Are both CBF1 and 2 needed to support stem cell growth? Or can this be achieved by only CBF1 or CBF2? Organoids would be a suitable model to examine this issue. According to the title, CBFs act as hubs to control colon homeostasis, what is not reflected in the experimental results.

As described in the previous reply, there is no clear marker gene(s) for CBFs1 vs. CBFs2 that show a non-overlapping expression pattern. Hence, the specific isolation and co-culture with intestinal organoids of these two populations is not currently feasible.

However, we respectfully disagree that our title does not reflect the current understanding of the likely role of CBFs. Excellent work by various groups has provided a solid basis for concluding that the expressed factors are required for colon homeostasis. Indeed, CBFs2 show higher but not exclusive expression of the Bmp antagonist *Gremlin1* and the Wnt potentiator *Rspo3*. In addition, both Gremlin1 (McCarthy et al., 2020) and Rspo3 (Greicius et al., 2018) have been shown to be able to substitute for Noggin and Rspo1, which is used in traditional organoid culture. CBFs1 on the other hand show higher but not exclusive expression of the canonical Wnt ligands *Wnt2* and *Wnt2b*. Wnt2b has been shown to support intestinal organoid growth (Farin et al., 2012).

Given that both Bmp antagonism and activity of canonical Wnt signaling is needed to support intestinal epithelial stem cells we conclude that both CBFs1 and CBFs2 are needed to support stem cells. Thus, we believe there is ample evidence to claim that CBFs and CTFs act as signaling hubs that control colon homeostasis.

These observations have been added to the manuscript when discussing the difference between CTFs1 and CTFs2. We believe the discussion prompted by the reviewers question is valuable.

Line 401: The statement "Canonical Wnt ligands, which are integral to intestinal epithelial stem cell maintenance, are secreted by epithelial Paneth cells in the small intestine" is misleading and should be corrected. Multiple studies from multiple labs have shown that Paneth cells, Wnt3 in intestinal epithelium, and Porcn and Wls in the intestinal epithelium are all completely dispensable for normal small intestine function.

Actually, this comment is pointing to the message we do not intend to portray. Our lab, as well as others previously published epithelial and mesenchymal cells to act as redundant sources of Wnt ligands in the small intestine. But to be clear, we changed the statement in the discussion accordingly.

Minor points:

Page 5, line 100: non-epithelial (EpCAM-, CD45+) cells should be ***CD45- cells***

Changed in manuscript.

Page 6, line 132: Dll1 and Dll4 expression is displayed in S1D not S1C

It is now corrected.

Page 6, line 135: GO-terms are shown in S1C not S1D

This is now corrected..

Page 6, line 137" Egf family ligands and ephrins expression can be found in S1D not S1C

We changed it in the manuscript.

Page 7, line 139: Ihh expression is shown in S1D not S1C

Corrected.

Sometimes the reference to the figures is missing in the text, what makes it difficult for the reader to follow the results, e.g.
- page 6, line 127: Lgr5, Olfm4, Axin2 and Mki67. Moreover, Olfm4 and Mki67 expression is not displayed in the figures

Reference added to the manuscript and the text was slightly changed. In addition, Mki67 expression is added to Fig1D.

- page 6, line 133: (Guaca2a+, Alpi+, Aldh1/1+)

Reference to respective figures are added in the manuscript.

Page 8, line 183: S1 and S1 should be S2

Changed in manuscript.

Page 10, line 233: Expression of Vimentin cannot be found in Fig. S3A

S4A Fig. depicts an antibody staining of Vimentin (Vim), showing co-expression of Vimentin protein and PdgfraEGFP.
